# Out of the Black Sea: Phylogeography of the Invasive Killer Shrimp *Dikerogammarus villosus* across Europe

**DOI:** 10.1371/journal.pone.0118121

**Published:** 2015-02-18

**Authors:** Tomasz Rewicz, Remi Wattier, Michał Grabowski, Thierry Rigaud, Karolina Bącela-Spychalska

**Affiliations:** 1 University of Lodz, Department of Invertebrate Zoology and Hydrobiology, Łódź, Poland; 2 Université de Bourgogne, Equipe Ecologie Evolutive, UMR CNRS 6282 Biogéosciences, Dijon, France; University of Windsor, CANADA

## Abstract

The amphipod *Dikerogammarus villosus* has colonized most of the European main inland water bodies in less than 20 years, having deteriorating effect on the local benthic communities. Our aim was to reveal the species phylogeography in the native Black Sea area, to define the source populations for the colonization routes in continental Europe and for the newly established UK populations. We tested for the loss of genetic diversity between source and invasive populations as well as along invasion route. We tested also for isolation by distance. Thirty three native and invasive populations were genotyped for mtDNA (COI, 16S) and seven polymorphic nuclear microsatellites to assess cryptic diversity (presence of deeply divergent lineages), historical demography, level of diversity within lineage (e.g., number of alleles), and population structure. A wide range of methods was used, including minimum spanning network, molecular clock, Bayesian clustering and Mantel test. Our results identified that sea level and salinity changes during Pleistocene impacted the species phylogeography in the Black Sea native region with four differentiated populations inhabiting, respectively, the Dnieper, Dniester, Danube deltas and Durungol liman. The invasion of continental Europe is associated with two sources, i.e., the Danube and Dnieper deltas, which gave origin to two independent invasion routes (Western and Eastern) for which no loss of diversity and no isolation by distance were observed. The UK population has originated in the Western Route and, despite very recent colonization, no drastic loss of diversity was observed. The results show that the invasion of the killer shrimp is not associated with the costs of loosing genetic diversity, which may contribute to the success of this invader in the newly colonized areas. Additionally, while it has not yet occurred, it might be expected that future interbreeding between the genetically diversified populations from two independent invasion routes will potentially even enhance this success.

## Introduction

Biological invasions are the inherent symptom of global changes and a major threat to biodiversity [1–3]. Alien species may cause irreversible changes to invaded ecosystems, often resulting in reducing distribution or in extinction of native species through direct predation [4], food and shelters competition [5, 6], transmission of parasites [7], or modifications of habitat [8].

Molecular markers proved to be powerful tools in tracking invasion patterns and dynamics [9, 10]. They were useful in identifying: (1) cryptic invasions, when one morphological invasive species is composed of at least two diverging evolutionary units [11–13] or when a species thought to be native appears to be an alien [14]; (2) source populations and pathways of introductions [15–18]; (3) diversity dynamics including either bottleneck or founder effects [19–21], or absence of diversity loss [22], or even diversity enrichment in newly established populations due to multiple introductions from different sources [18, 23]; (4) hybridization, often associated with enhanced invasiveness [24–26], involving multiple introduction sources [17, 27], hybridization between native and invasive species [28, 29] or introgression [30].

Such information is still scarce in case of some prominent invaders. One example is the amphipod *Dikerogammarus villosus* (Sowinsky, 1894), also known as the ‘killer shrimp’, which colonized most of the European main inland waters in less than 20 years [31–33]. This species is an efficient, high trophic level predator [34–37], characterised by a wide ecophysiological tolerance [38–41] as well as by a very high fecundity [42–45].

**Fig 1:**
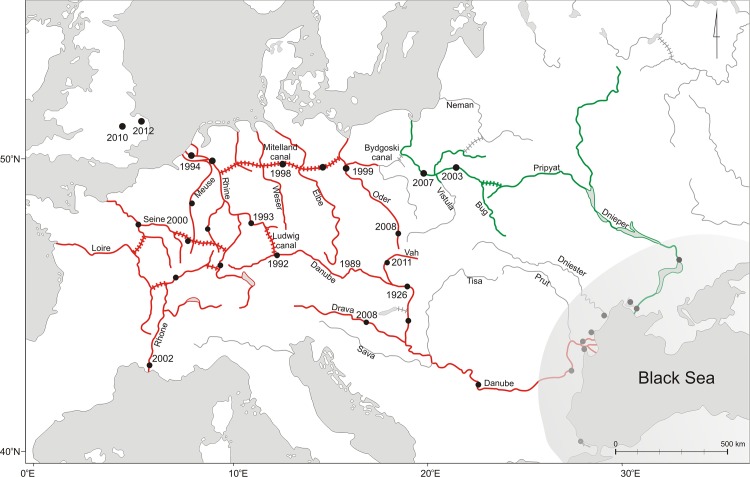
Distribution of *Dikerogammarus villosus* in its native (N) area in the Black Sea basin (shaded area) and along Eastern (E) and Western (W) routes (R) of invasions in continental Europe as well as in UK. Dashed lines represent canals. The presumed Western Route is indicated in red, Eastern Route in green. Numbers are dates of the first report of *D. villosus* at these sites. Black dots are sampling sites used in this study.

Occurrence of the species in its native area is associated predominantly with brackish lagoons (limans) and lower reaches of large rivers draining to the Black Sea. Its phylogeographic history in the native area is unknown, although it could bring key information for understanding current invasion dynamic, as in the case of other Ponto-Caspian intruders [46,47]. The distribution in the invaded continental Europe was a subject of numerous studies and is well documented (summarised in Rewicz et al. [48]). Based on the distribution pattern, two major distinct routes for the invasion of *D. villosus* have been proposed [49]. The eastern route would encompass Dnieper, Prypiat, Bug and Vistula rivers (Fig. 1). The western one would be composed of the Danube, Rhine, main French rivers, but also some northern sites of central Europe, such as the Mittelland Canal in Germany, and Oder in Poland (Fig. 1). However, the existence of these two distinct routes has not been firmly tested, and numerous points are still subjects of a debate.

First, the origin and genetic diversity of populations found in northern and central Europe (i.e. Oder River and Mittelland Canal) is not clear. Colonization of these sites could be a westward expansion of the eastern route, from the Bug and Vistula rivers, based on the fact that other Ponto-Caspian invasive species followed such direction [49]. However, *D. villosus* has not been found in the waterways joining the Vistula and the Oder and the dates of first records of *D. villosus* from both river systems suggest that it was present in the Oder prior to colonization of the Vistula and Bug rivers [49]. Therefore, a secondary eastward extension of the western route was favored to explain *D. villosus*’s presence in northern Germany and western Poland. If correct, there would be two fronts of invasion in Poland (eastern and western), presently 150 km apart and likely to get in contact in the near future [31].

These two fronts might be genetically distinct. First, they originated from different parts of the native area (Fig. 1). Second, genetic differentiation between source populations of Ponto-Caspian species is already known for other invaders, such as mysids [50] and gobies [47].

A second question is the global impact of the invasion process on the genetic variation of *D. villosus*. So far, only three studies dealt with its genetic diversity. They focused either on molecular identification of *D. villosus* versus two congeneric species present in the Danube [51,52] or on its invasion dynamics in south-western Europe [53]. The latter study suggested there was no loss of genetic diversity during the invasion process. However, this study was based on few molecular markers, and it is unknown if this pattern restricts to the western route of colonization, or if it is a general pattern for *D. villosus* invasion. In particular, genetic variation within the eastern route has not yet been explored. Additionally, the genetic variation of the populations recently established in UK [54], as well as their source, are unknown.

In this study, *D. villosus* populations from the western part of the Black Sea basin (native area), as well as invasive populations from the presumed western and eastern colonization routes and from UK were genotyped for mtDNA (COI, 16S) and seven polymorphic nuclear microsatellites, in order to answer the following questions: (1) What is the species phylogeography in the native Black Sea area, including the assessment of cryptic diversity (presence of deeply divergent lineages), historical demography, level of diversity and genetic differentiation between populations being potential sources for the two presumed colonization routes? (2) Is it possible to associate a distinctive genetic signature to the two presumed colonization routes in continental Europe? (3) Is there a loss of genetic diversity between source populations and colonized areas, and is there a loss of genetic diversity and an isolation by distance along the colonization routes? (4) What was the source(s) for the newly-established UK populations and was this colonization associated with a genetic bottleneck?

## Materials and Methods

### Sample collection

*Dikerogammarus villosus* was collected from 33 sites, both in the native area (hereafter N) and the invaded part of Europe (Fig. 1, Table 1), during expeditions spanning 2002–2012. All the sampling sites were located in public and non-protected areas. No permissions were required for sampling. The study did not involve any endangered or protected species. In the native area, all suitable coastal habitats were surveyed along the western and northern coast of the Black Sea. In the invaded continental Europe, sampling covered both the putative western and eastern routes (hereafter WR and ER, respectively) and the recently invaded UK. One site in UK was sampled twice, in 2010 and 2012.

**Table 1:** Sampling sites of *Dikerogammarus villosus*.

Site	Acronym	River Basin	River	Date	Co	Latitude	Longitude	mtDNA	msat
1	N	Dniester	Dniester	2009	UA	46.25705	30.41911	24	32
2	N	Durungol*	Durungol*	2007	TR	41.3163	28.62055	23	32
3	N	Dnieper	Dnieper	2009	UA	46.60276	32.58274	18	32
4	N	Dnieper	Dnieper	2009	UA	47.79173	35.12568	13	32
5	N	Dnieper	Dnieprovsky*	2011	UA	46.61579	32.09658	11	0
6	N	Danube	Danube	2011	UA	45.33713	28.95544	12	31
7	N	Danube	Kunduk lake	2011	UA	45.54009	29.65501	12	32
8	N	Danube	Danube	2002	RO	45.180576	28.804091	10	0
9	N	Danube	Danube	2002	RO	44.409714	27.88395	10	0
10	ER	Vistula	Bug	2006	PL	52.265379	23.181946	13	32
11	ER	Vistula	Vistula	2008	PL	52.384203	20.186637	5	32
12	WR	Danube	Danube	2011	RO	43.9955	22.92567	12	32
13	WR	Danube	Danube	2002	HU	46.623749	18.865837	6	0
14	WR	Danube	Drava	2011	HR	46.17702	17.00734	12	32
15	WR	Danube	Danube	2011	HU	47.785567	18.959883	11	32
16	WR	Danube	Vah	2011	SK	48.9757	18.15061	10	32
17	WR	Danube	Danube	2002	DE	48.915473	11.880207	6	31
18	WR	Rhein	Main	2002	DE	49.794246	9.927511	7	32
19	WR	Rhein	Rhein	2008	FR	47.819856	7.541625	6	32
20	WR	Rhein	Mosel	2002	FR	49.199649	6.200584	6	0
21	WR	Rhein	Mosel	2002	FR	48.681174	5.903514	6	32
22	WR	Meuse	Meuse	2002	FR	50.049442	4.722132	6	31
23	WR	Rhein	Ijssel	2010	NL	52.2388	6.15999	12	23
24	WR	Amstel	Ijmeer	2002	NL	52.394057	5.151378	6	32
25	WR	Seine	Seine	2002	FR	47.101059	5.26419	5	31
26	WR	Seine	Marne	2002	FR	48.821061	2.4697	6	31
27	WR	Rhone	Rhone	2002	FR	43.813934	4.646806	6	30
28	WR	Weser	Mittelland canal	2010	DE	52.38907	9.35703	12	24
29	WR	Elbe	Mittelland canal	2010	DE	52.41572	12.49422	12	20
30	WR	Oder	Oder	2008	PL	52.496313	14.640777	12	31
31	WR	Oder	Oder	2009	PL	50.411732	18.107727	11	24
32A	UK	Great Ouse	Grafham Water	2010	UK	52.291832	-0.32	12	32
32B	UK	Great Ouse	Grafham Water	2012	UK	52.291832	-0.32	9	32
33	UK	Norfolk Broads	Norfolk Broads	2012	UK	52.739245	1.496202	8	25

N, Native Black Sea area; ER and WR, Eastern and Western Route; UK, United Kingdom. See Fig. 1 for details about geographic distributions of sites.

UA, Ukraine; RO, Romania; TR, Turkey; PL, Poland; HU, Hungary; HR, Croatia; SL, Slovakia; DE, Ger; FR, France; NL, Netherlands and UK = United Kingdom. *, Liman.

Acronyms (explanation below the table); Co, Countries; Decimal coordinates; mtDNA and msat: sampling size for mtDNA and microsatellite markers, respectively.

### Molecular analysis

DNA from 876 samples was extracted with a standard phenol-chloroform method after Hillis et al. [54]. Air-dried DNA pellets were eluted in 100 μl of TE buffer, pH 8.00, stored at 4°C until amplification, and subsequently at -20°C long-term storage. A total of 350 specimens were amplified for two mtDNA markers: 16S ribosomal RNA (16S rRNA; ca. 320 bp fragment) with LR-J-GAM/LR-N-GAM primers [51] and reaction conditions following Grabowski et al. [14] and Cytochrome Oxydase subunit 1 gene (CO1; ca. 670 bp fragment) with LCO1490/HCO2198 primers [55] and reaction conditions following Hou et al. [56]. Sequences were obtained using BigDye sequencing protocol (Applied Biosystems 3730xl) by Macrogen Inc., Korea. Sequences were edited and aligned with ClustalW algorithm [57] using BioEdit© 7.2.5, leading to 350 sequences of 16S (303 bp) and COI (654 bp) which were concatenated to perform analyses. Haplotypes were retrieved using DnaSp v5 both for individual markers and concatenated data [58]. Haplotypes for individual markers were deposited in GenBank (accession numbers: KM208862-KM208879).

Seven microsatellite loci (msat) were used as nuclear co-dominant molecular markers: DikS, DikF [52], Dv11, Dv13, Dv17, Dv31, Dv33 [59]. A total of 876 specimens were genotyped, locus DikF amplified only for Danube and Western Route. PCR conditions were described by Wattier et al. [52] and Rewicz et al. [59]. Microsatellite alleles were visualized in 6.5% acrylamide 25 cm long gels on a LICOR 4200 L automated sequencer and scored by eye. Reference individuals were included for inter-gel calibration.

### Testing for cryptic diversity

To visualize molecular divergence of mtDNA haplotypes, a Minimum Spanning Network was generated using Arlequin 3.5.1.2 [60]. Pairwise Kimura 2 parameter (K2p) distances were estimated using Mega 6.2 [61]. For analysis based on Bayesian inference, the AICM method of moments’ estimator [62] was used to define best fitting model of evolution. The time calibrated phylogeny was reconstructed in BEAST, version 1.8.1 [63]. The Hasegawa, Kishino and Yano (HKY) model of evolution with proportion of invariable (I) and Yule speciation model were set for priors. The strict clock with rate 0.0142 proposed for the genus *Gammarus* was applied for the analyses [64]. Two runs of 20 M iterations of Markov chain Monte Carlo (MCMC) sampled each 1000 iterations were performed. Both runs were examined using Tracer v 1.6, all sampled parameters achieve sufficient sample sizes (ESS>200). Tree files were combined using LogCombiner 1.8.1 [63], with removal of the non-stationary 10% burn-in phase. The maximum clade credibility tree was generated using TreeAnnotator 1.8.1 [63]. To add additional support for the tree topology, the same dataset was analyzed with Maximum Likelihood (ML) method based on the General Time Reversible (GTR) model [65] with 10000 bootstrap replicates. Model of evolution was selected using jModelTest2 [66]. ML analyses were performed in the MEGA 6 [61].

### Historical demography within the native range based on mtDNA

To reveal historical demography in the Ponto-Caspian region we used 133 individuals from nine localities (Table 1). In order to assess the temporal changes of the effective population size in each of the three phylogeographic lineages (A-C, see results), a set of the Extended Bayesian Skyline Plot (EBSP) analyses [67] was performed in BEAST, version 1.8.1 [63]. The GTR model of evolution was used as the best fitting model. To ensure convergence, four runs of MCMC, 100M iterations long sampled each 1000 iterations, were performed. Both runs were examined using Tracer v 1.6, all sampled parameters achieved sufficient sample sizes (ESS>200).

### Allelic/haplotypic diversity and differentiation

Diversity was assessed by calculating: (1) allelic-haplotypic (msat/mtDNA) diversity (k), (2) allelic richness (*A*_r_) and private allelic richness (*PA*_r_) corrected for a common sampling size using rarefaction approach [68]. Calculations were performed with Hp-Rare 1.1 [69], differentiation in *A*_r_ was tested using the non-parametric Mann-Whitney U-test in Statistica 10 [70], and put in brackets if significant. In addition, observed heterozygosity (*H*_O_), expected heterozygosity (*H*_E_) and fixation index (*F*_IS_) were calculated, when appropriate, for microsatellite markers using Fstat [71]. Pairwise differentiation was determined by two *F*_ST_ estimators: Θ_ST_ with Tamura-Nei distance for mtDNA [72] and Θ for microsatellites [73], both implemented in Arlequin, statistical significance being measured using 10000 permutations. Genetic diversity and *F*_ST_ were assessed either pooling sampling sites, or not, according the hypothesis tested, e.g. between fronts in Poland.

Population structure was also analyzed using individual-based Bayesian clustering method implemented in Structure 2.3.4 [74]. Simulations were performed on the full data set including 29 populations and 876 individuals. Runs for each possible value of *K* (1 to 8) were repeated 20 times. Each run used a burn-in of 500000 iterations, a run length of 750000 iterations. All simulations were performed using the admixture and correlated allele frequencies models with no prior information. Selection of most probable value of *K* relied on the Δ*K* method developed by Evanno et al. [75].

### Diversity and differentiation along Western Route (WR)

Based on 20 sites along the WR we tested if microsatellite differentiation increased positively with distance between sites (isolation-by-distance, hereafter IBD) but also if diversity (mean allelic richness) was associated with geographical distance from the source area (Danube delta). The distances were estimated using Google Earth v.7.1.2. IBD was tested using Mantel test between *F*_ST_ / (1- *F*_ST_) and geographic distance as recommended by Rousset [76] for testing IBD in one-dimensional linear systems, with 100000 permutations, using the GenePop on the Web 4.2 [77] and ISOLDE software.

## Results

### Phylogeography in the native Black Sea area

**Fig 2:**
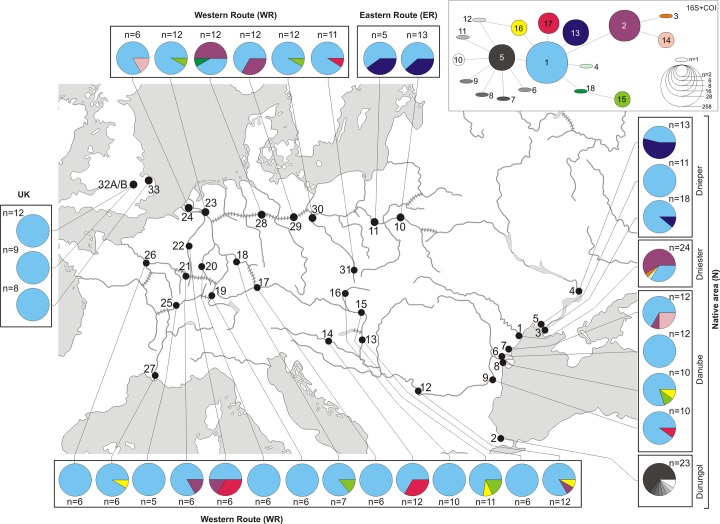
Geographical distribution of *D. villosus* haplotypes in the native and invaded area. Numbers near black circles represent sampling localities coded as in Table 1. In upper right corner is the minimum spanning network of mtDNA haplotypes identified in *D. villosus*.

**Fig 3:**
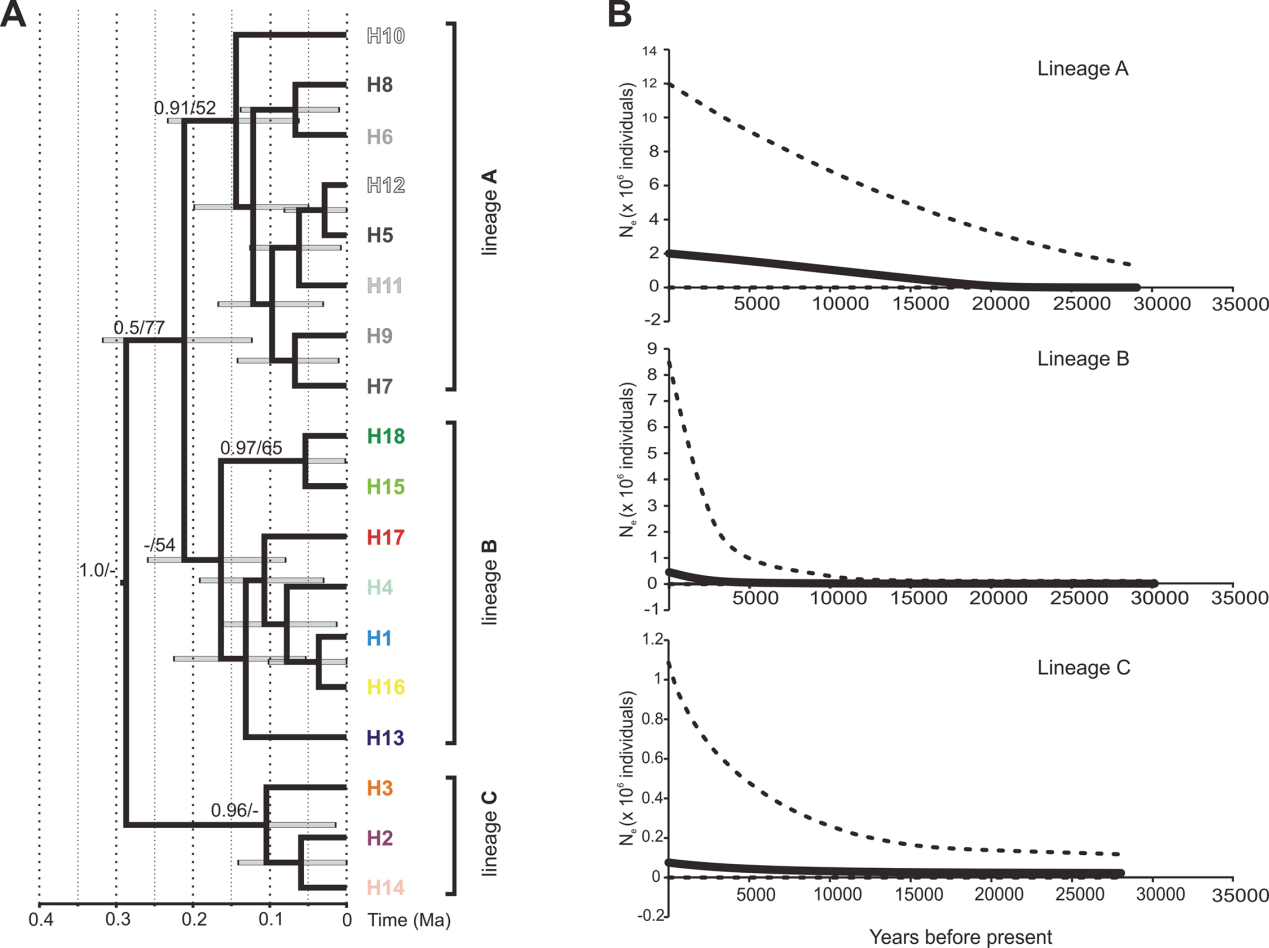
Phylogeny and demographic history of *D. villosus* in the native Black Sea area. (A) Maximum clade credibility chronogram inferred from a strict molecular clock model based on the concatenated COI+16S data set of *Dikerogammarus villosus*. The two numbers given next to the respective node indicate Bayesian posterior probabilities (> 0.5), and maximum likelihood bootstrap values (> 50%). (B) Multilocus extended Bayesian skyline plots for three linages of *Dikerogammarus villosus*. Solid lines indicate the median posterior effective population size through time; dashed lines indicate the 95% highest posterior density interval for each estimate.

Out of 133 individuals from 9 sites in the native Black Sea region, a total of 17 haplotypes were identified based on concatenated (303+654 bp) 16S and COI mtDNA sequences (S1 Table). First, the difference observed between the most divergent haplotypes was only five nucleotides (Fig. 2). Second, the mean overall K2p genetic distance between haplotypes was very low being 0.0009 (SD 0.0004). It showed clearly that there is no cryptic diversity involving highly divergent lineages. However, combination of the haplotype network (Fig. 2) and Bayesian phylogenetic reconstruction (Fig. 3A) revealed that the haplotypes may be grouped into three phylogenetic lineages. Their spatial distribution is partly structured geographically. Lineage A includes eight haplotypes (5–12) specific to the Durungol liman in Turkey (2-N). Lineage B includes 7 haplotypes (1, 4, 13, 15–17) and lineage C includes the 3 remaining haplotypes (2, 3 and 14). The Bayesian chronogram showed that C diverged from A+B ca 280 kyr BP, while A and B split ca. 200 kyr BP (Fig. 3A). The results of the EBSP analysis indicated that population of *D. villosus* in the Durungol liman experienced steady growth for the last 20 ky, while populations of the remaining two lineages in the native area remained stable for most of the last 30k years, with accelerated growth starting less than 10 ky ago (Fig. 3B).

The highest diversity for mtDNA and msat was observed in Durungol (*A*_r_ = 5.3 and 5.73 respectively); the locality harboring also the highest private allelic richness (*PA*_r_ = 5.3 and 1.08) (Table 2). The potential (i.e. *a priori*) source areas of invasion, i.e. Dnieper and Danube deltas, did not exhibit the same level of mtDNA diversity (*A*_r_ = 2 and 3) but harbored some specific haplotypes (Table 2, Fig. 2). For msat these areas had similar diversities (*A*_r_ = 3.94 and 3.57) and low private allelic richness (*PA*_r_ = 0.05 and 0).

**Table 2:** Genetic diversity for *Dikerogammarus villosus* calculated for microsatellites (msat) or mitochondrial DNA (mtDNA) within sampling sites or groups of sites.

Sites	Acronym	msat				mtDNA			
		n	K	*A*_r_	*PA*_r_	n	K	*A*_r_	*PA*_r_
1	N	32	5.33	5.07	0.45	24	4	3.1	1.8
2	N	32	6.17	5.73	1.08	23	8	5.3	5.3
3+4+5	N	64	4.83	3.94	0.05	42	2	2.0	0
6+7+8+9	N	63	4.00	3.57	0.00	44	6	3	0.7
10+11	ER	64	3.83	3.56	0.00	18	2	2.0	0
30+31	WR	55	3.67	3.37	0.00	23	3	2.2	0.5
23+24	WR	55	4.17	3.8	0.10	18	3	2.40	0.4
32A	UK	32	3.71	3.57	0.00	12	1	1	0
32B	UK	32	3.57	3.49	0.00	9	1	1	0
33	UK	25	2.71	2.49	0.00	8	1	1	0

See Table 1 for site and acronym definition. n, number of individual analyzed; K, average number of alleles or haplotypes; *A*_r_ and *PA*_r_, allelic and private allelic richness estimated with correction for sample size through rarefaction, for msat and mtDNA respectively.

**Fig 4:**
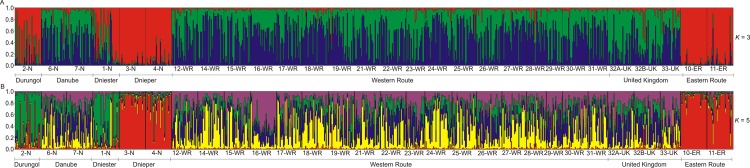
Bayesian clustering of *Dikerogammarus villosus* based on six microsatellite loci in 29 populations collected in native and invaded area with *K* = 3 (A); and *K* = 5 (B). Acronyms refer to sites as explained in Table 1. Each individual is represented by a thin vertical line, with proportional membership in different clusters indicated by colors. Black vertical lines separates sampling sites, with site identification indicated below the plot.

Genetic differentiation differed from zero for all area pairwise comparisons, for both msat and mtDNA data (Table 3). However, the level of differentiation was heterogeneous, being the highest between Durungol and Dnieper (*F*_ST_ = 0.180 and Θ_ST_ = 0.693) and the lowest between Danube and Dniester (*F*_ST_ = 0.048 and Θ_ST_ = 0.103). The results of Bayesian clustering suggest that the four selected areas may represent four genetic clusters, although the division is not strict. The Durungol and Dnieper populations are the most homogeneous ones, while the Dniester and Danube populations show symptoms of migration or very recent common ancestry (Fig. 4A,B).

**Table 3:** Genetic pairwise differentiation for *Dikerogammarus villosus* estimated for microsatellites (*F*_ST_, below diagonal) or mitochondrial DNA (Θ_ST_, above the diagonal) between sites or group of sites in the native area and invasion fronts in Poland.

Sites	Acronym	*F*_ST_\Θ_ST_					
		1	2	3+4+5	6+7+8+9	10+11	30+31
1	N	-	0.657***	0.561***	0.402***	0.506***	0.516***
2	N	0.127***	-	0.693***	0.606***	0.652***	0.679***
3+4+5	N	0.133***	0.180***	-	0.103***	0.039ns	0.111*
6+7+8+9	N	0.048***	0.122***	0.157***	-	0.198***	0.011ns
10+11	ER	0.157***	0.158***	0.027***	0.165***	-	0.274***
30+31	WR	0.089***	0.153***	0.215***	0.019***	0.202***	-

ns, not significant

*P ≤ 0.05

***P ≤ 0.001

See Table 1 for site and acronym definition.

### Colonization dynamics in Continental Europe

For mtDNA, ER and WR in Poland, i.e. fronts, are not differentiated from their respective putative sources in the native region, i.e. Dnieper (3+4+5) and Danube (6+7+8+9), respectively (Table 3), while differentiation between fronts was significant (Table 3). For msat, although all pairwise comparisons for differentiation were significant (Table 3), the level of differentiation between fronts in Poland and their respective putative areas of origin was low (*F*_ST_ = 0.027 and = 0.019) compared to differentiation among sites belonging to different routes (0.157 < *F*_ST_ < 0.215) (Table 3). Bayesian clustering analysis showed clearly, that individuals from the western front (Poland), the western route and the source in Danube from a homogeneous genetic unit, while the eastern front with its putative source in Dnieper form another homogeneous genetic unit (Fig. 4A,B).

**Fig 5:**
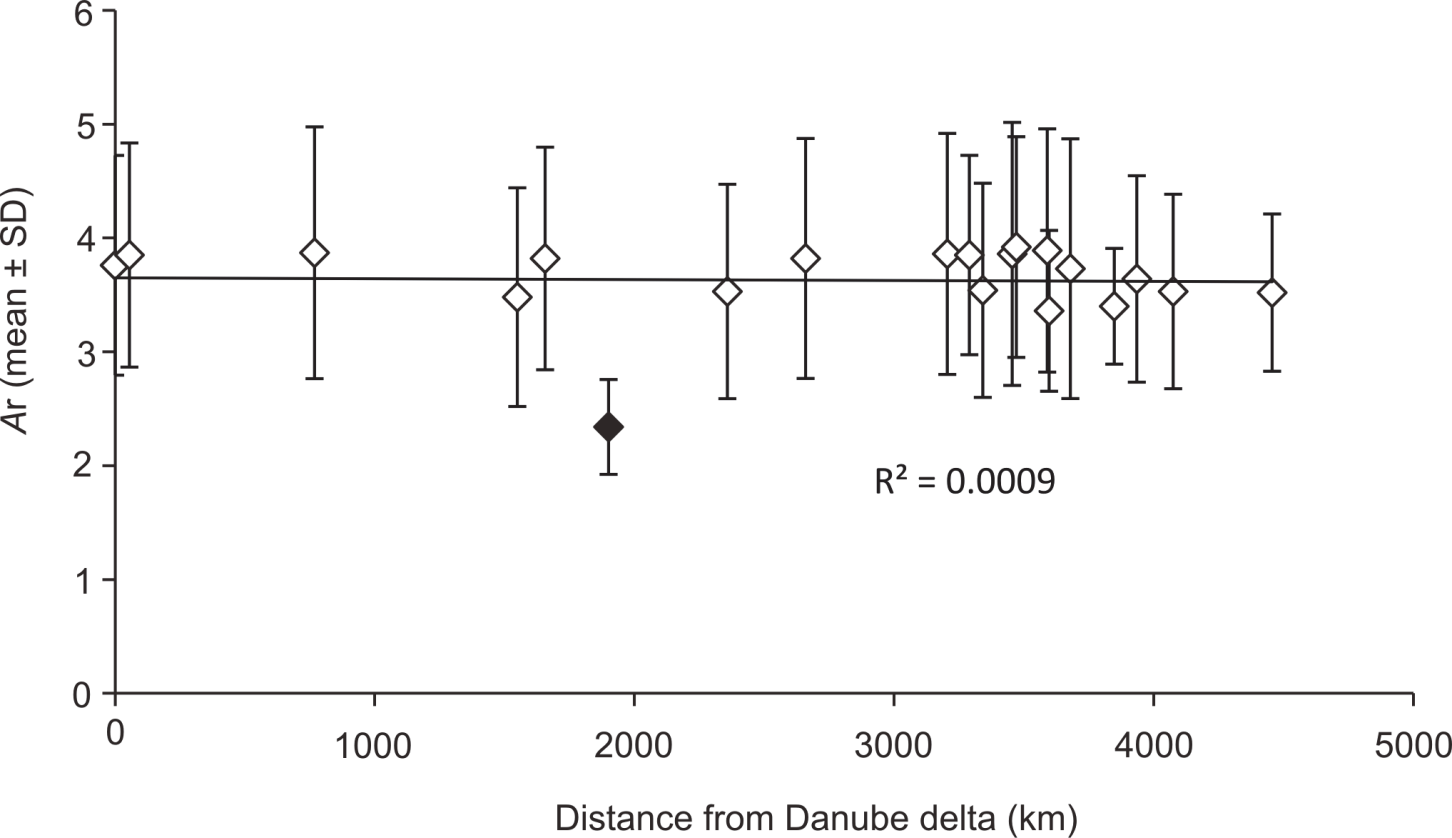
Allelic richness across seven microsatellite loci (mean ± standard deviation) within 20 populations of *Dikerogammarus villosus* from Western Route (WR) plotted against linear distance from Danube delta to each site along the route (see Fig. 1. for details). Black diamond indicate Vah river site (site 16), see text for details.

**Fig 6:**
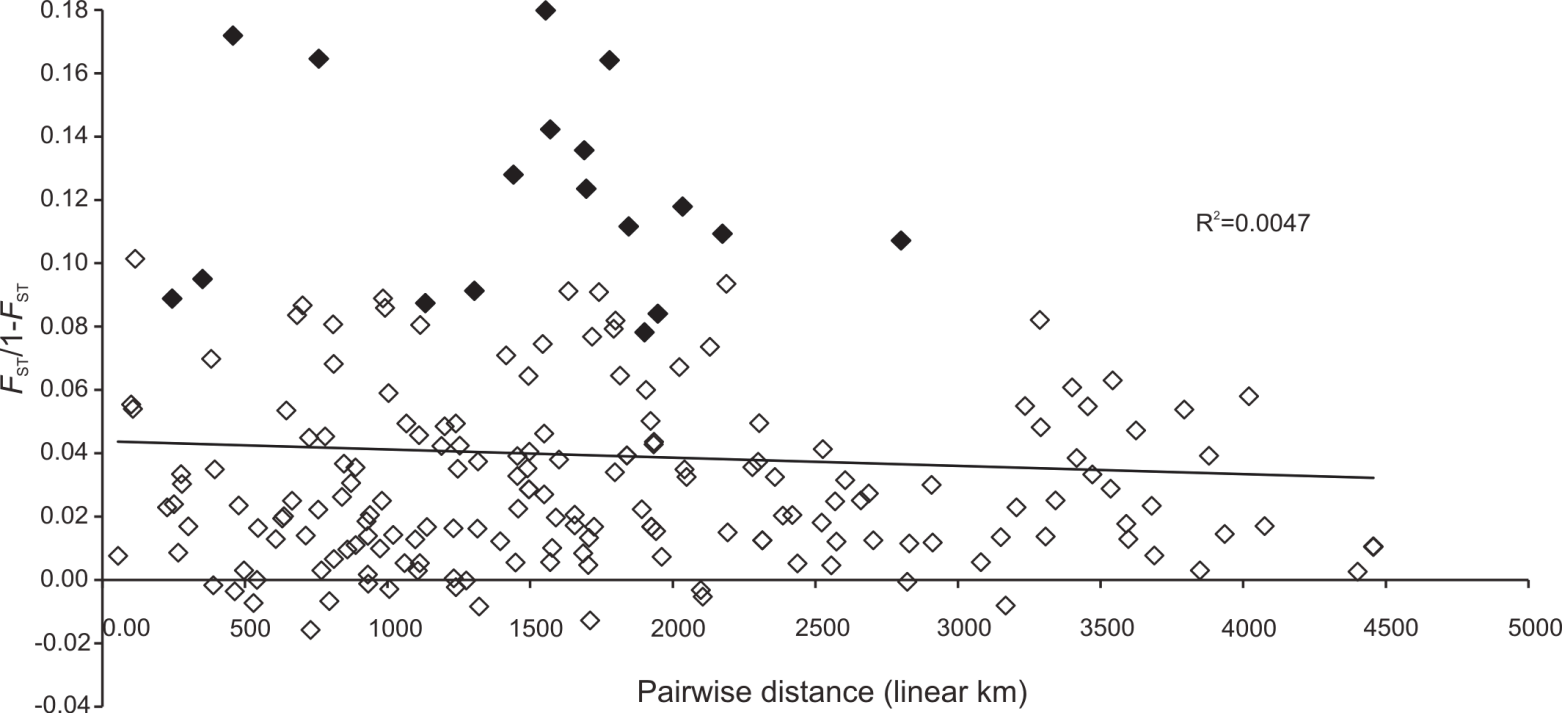
Plot of pairwise *F*_ST_ (seven microsatellite loci) versus pairwise linear distance of 20 populations of *Dikerogammarus villosus* encompassing source populations for the Western Route (WR), the WR itself and populations in the western front in Poland. Black diamonds highlight pairwise comparisons with Vah river site (site 16), see text for details.

Both fronts in Poland had the same level of diversity for msat compared to their putative source in the native region i.e. in Dnieper and Danube respectively (Table 2). This is also true for mtDNA for beginning the end of the ER, but the WR in Poland seems to present less diversity than the Danube (6+7+8+9) (Table 2). Along the WR, geographic distance from the source area did not explain msat diversity within sites (Fig. 5). Along the 4500 km long route, only one site located on the River Vah in Slovakia (site 16) had lower diversity. No isolation by distance (Fig. 6) was present as no significant correlation was detected between pairwise geographical distances and genetic differentiation (Mantel test, R^2^ = 0.0047, *P* = 0.42).

### Source population and diversity for the UK

We observed high genetic differentiation for both mtDNA (0.206 < Θ_ST_ < 0.298) and msat (0.138 < *F*_ST_ < 0.172), for pairwise comparisons between ER in Poland and two pooled sites in the Netherlands (23+24) or any UK site. On the opposite, pairwise comparisons between two sites in the Netherlands and each UK site showed no significant differentiation for mtDNA (Table 4). For msat, lower level of differentiation was observed between the UK site 32 (A and B) and the Netherlands (0.026 < *F*_ST_ < 0.035) than between the UK site and ER in Poland (0.138 < *F*_ST_ < 0.172). The UK site 33 showed a less conclusive picture for *F*_ST_ (Table 4). Bayesian clustering analysis (Fig. 4A,B) showed that the UK populations form a homogeneous genetic unit with the western route, and genetically different from the eastern route.

**Table 4:** Genetic pairwise differentiation estimated for microsatellites (*F*_ST_, below diagonal) or mitochondrial DNA (Θ_ST_, above the diagonal) between sites or group of sites in the possible source populations for UK and UK sites.

Sites	Acronym	*F*_ST_\Θ_ST_				
		10+11	23+24	32A	32B	33
10+11	ER	-	0.206*	0.298*	0.264*	0.250*
23+24	WR	0.139***	-	-0.024ns	-0.044ns	-0.053ns
32A	UK	0.172***	0.026***	-	0.000ns	0.000ns
32B	UK	0.138***	0.035***	0.027***	-	0.000ns
33	UK	0.172***	0.096***	0.117***	0.124***	-

ns, not significant

*P ≤ 0.05

***P ≤ 0.00

See Table 1 for site and acronym definition.

Only one mtDNA haplotype (haplotype 1), the most common in continental Europe, occurred in UK. Haplotype 4, while common in ER, was absent from the UK. In Grafham Water site (32A, 32B) we observed no loss of diversity for msat compared to the Netherlands with *A*_r_ values being respectively 3.57 and 3.49, versus 3.8 (Table 2). Diversity in the more recent population (33) was 2.49 but did not differ in the statistical terms from the above *A*_r_ values (Table 2).

## Discussion

The Ponto-Caspian region has been recognized as the most prominent donor of non-indigenous hydrobionts to Europe and to the North American Great Lakes system. Their taxonomic spectrum is wide including amphipods, mysids, cladocerans, gastropods and fishes [79–82]. Phylogeography and population genetics patterns of these invaders may help in understanding colonization dynamics and in controlling their further spread [16]. Our results confirm that the invasion scheme for one of these species, *D. villosus*, is complex, with multiple routes, and that the loss of genetic diversity during the course of colonization is weak. We evidenced that colonization of the UK was originating from one (here Western) out of the two genetic units associated with continental invasion.

### Phylogeography and contemporary genetic structure in the native region

Level of cryptic diversity for invertebrates in the Ponto-Caspian region is highly variable. Deep level of divergence, but below the species threshold, was observed for e.g. cladocerans [78] and mysids [79]. Cryptic diversity was revealed e.g. in monkey goby [80]. Although the phenomenon is known to occur in several amphipods [81, 82], no cryptic diversity was detected in *D. villosus* in its native region. The 17 mtDNA haplotypes showed shallow divergence with an overall K2p genetic distance of 0.0009, far below the threshold of 0.03–0.055 identified between crustacean species [83]. Such low divergence may be related to very recent history of the species within the Black Sea region (see below).

Although shallow, the divergence between mtDNA haplotypes is geographically structured with lineage A including a set of 8 haplotypes (out of 17) and being restricted to the Durungol liman. This pool of haplotypes separated from others ca 200 kyr BP. In addition, the other sampled areas are also characterized by high private haplotypic richness. Overall, the level of differentiation (Θ_ST_) between populations is high in the Black Sea area. The turbulent Pleistocene hydro-geological history of the region with recurrent changes of sea level and salinity may be among the most powerful driving forces explaining this pattern [46, 50, 78, 79, 84–86]. During the last 670 kyr, there were at least 12 significant saline water intrusions from the Mediterranean Sea, and eight intrusions from the Caspian Lake to the Black Sea [87]. These events caused water level fluctuations and substantial salinity shifts from nearly fresh to full marine conditions that could cause shift ranges and population fragmentations in oligohaline hydrobionts inhabiting this basin [88]. During fully marine salinity stages, slightly brackish estuaries and limans may have become isolated refugia and differentiation centers for local aquatic fauna. The dating of divergence between Durungol liman and others sites coincides with one of the most prominent salinity raises [87]. In other sites presence of shared haplotypes reflects probably both recent and historical migration events among various areas in the native region. However, the overall presence of private haplotypes and high differentiation level indicate possible founder effects at the time of colonization. It could be followed either by restricted gene flow (which is confirmed by msat results) or even by allopatric divergence during stages of raised salinity. The results of EBSP analyses support very recent post-Pleistocene demographic expansion, suggesting that ecological conditions were locally favorable over evolutionary time-scale. Phylogeographic structure was already observed in mtDNA of Ponto-Caspian mysids [50, 79, 84], cladocerans [78] and gammarids [46, 78, 86].

The msat analyses pointed out high level of differentiation (*F*_ST_) between the four native sampled areas, that were divided in four genetic clusters in Bayesian analyses. If the Durungol liman is clearly isolated from the other sampled areas, the latter show connectivity as pointed out by unclear Bayesian assignment of some individuals. One of the explanations can be a shorter geographic distance between these populations. The north-western Black Sea is also the shallowest and least saline, due to massive sedimentation and inflow of riverine waters from e.g. Dnieper and Danube rivers [89, 90]. Chances for migrations between these populations are high, including anthropogenic transport due to the high ship traffic between local ports.

### Invasion routes and dynamics in continental Europe

In our study, combination of mtDNA and msat analyses clearly identified Danube and Dnieper deltas as differentiated sources for the two invasion routes we named “Western” and “Eastern”, respectively. *Dikerogammarus villosus* has been highly monitored throughout Europe due to its detrimental impact on the ecosystem. Therefore, accurate map of invasion progress can easily be drawn and converted into the most likely scenario for colonization routes [33, 89–92]. Agreement of our results with putative routes might seem trivial at first sight. However, few studies upon other species pointed out that molecular data identified routes that were different from the most likely, census-based, scenarios [9, 93]. Based on geographic invasion patterns of several aquatic species, Bij de Vaate et al. [91] defined three invasion corridors from the Ponto-Caspian region into continental Europe i.e. the northern (Volga River, Beloye, Onega and Ladoga lakes, Neva River to the Baltic Sea), the central (Dnieper, Pripyat, Pripyat-Bug channel, Vistula, Oder, Mittelland canal) and the southern (Danube, Rhine) one. Numerous species invasions fitted this pattern [49, 94]. Contrary to other species, *D. villosus* used only the eastern part of the central corridor and has not passed the Bydgoski channel in Poland which is connecting the Vistula and the Oder rivers (Fig. 1). On the other hand, the western part of the central corridor was colonized eastwards by population which came up the entire southern corridor westward. Possibly, the Bydgoski channel, with its prominently soft bottom, slow current and abundant vegetation is not prone to be colonized by *D. villosus* [95]. However, the closely related invader, *D. haemobaphes*, along with some other invasive amphipods, such as *Echinogammarus ischnus* and *Chelicorophium curvispinum*, managed to pass the Bydgoski channel and use the entire central corridor westwards. This channel was an important shipping route until mid-20th century, but the traffic now is heavily limited [96]. Yet, we cannot exclude possibility of future contact between these two distinct populations of *D. villosus*. The two fronts in Poland are differentiated and characterized by level of diversity analogous to their source regions. If the two fronts meet, hybridization will occur as the two populations are not phylogenetically and ecologically divergent which implies the absence of reproduction barrier. This may result in producing a potential “super-hybrid” – an even more effective invader, as it was observed in other cases [25, 26]. Thus, the situation deserves particular surveillance and management to avoid contact between these two fronts.

The WR has a length of about 4500 km from the source population in the Black Sea region to the invasion front in Poland. All mtDNA haplotypes found in the native range were observed in the invaded area, 3 out of 8 non-frequent haplotypes being present in the last 1000 km. In addition, in the Mittelland Canal we found one haplotype (haplotype 18) not even encountered in the native area, probably due to its very low frequency. For microsatellites, we found no loss of diversity along the route and no isolation by distance. Globally, it suggests that no bottlenecks occurred along WR. Similar conclusions were made by Müller et al. [51] and Wattier et al. [53], who conducted research on a smaller scale or with fewer genetic markers. Even if reduction of diversity in the invaded areas was often expected in the literature in 20th century, numerous studies since then have shown it might be far from being the rule [97–99]. Lack of diversity loss may result from a very large propagule size i.e. large founding population, and/or propagule frequency i.e. recurrent waves of invaders. We suspect the latter to play an important role for *D. villosus*. Indeed, no loss of diversity is observed while genetic differentiation between sites is present. This suggests that recurrent waves are both maintaining allelic diversity at a high level, and reshuffling allelic frequencies what generates differentiation.

Only one site (site 16) in the middle section of River Vah, was characterized by both low diversity and high level of differentiation from other sites. Strong founder effect is likely to explain this pattern as, probably, the site was sampled very recently after *D. villosus* first colonization [48, 100].

### Recent UK overseas conquest

The overseas introduction of *D. villosus* into UK in 2010 was noticed in popular media [54]. It proved clearly that large funds spent on biosecurity programs to prevent the spread of invasive species (i.e. the procedure “check, clean and dry”) have been insufficient to stop the killer shrimp [101, 102]. Both *F*_ST_ and Bayesian clustering of msat allowed us to exclude the populations from ER as donors for the UK sites. In addition, the haplotype 4, both frequent and private to ER, was not detected in UK, although our sampling size was limited in this case.

Diversity in any UK population was not different from the continent and no bottleneck effect was observed. Apparently, the propagule pressure was high enough to alleviate diversity loss. We are not able to conclude whether the introduction to UK was a single event followed by secondary colonization or multiple introductions. Anyway, the killer shrimp is spreading very efficiently throughout the UK. Furthermore, another congeneric invader, the “demon shrimp”, *D. haemobaphes*, has already been recorded in UK [103]. Based on several possible expansion models of both *D. villosus* and *D. haemobaphes* it has been estimated that more than 60% of the UK waterbodies is suitable and vulnerable to colonisation by these two invaders [101, 104–107]. Moreover, high popularity of water sports may further accelerate the invasion [108], due to high ability of the killer shrimp to spread via boating and diving equipment [32].

### Conclusions

Our results identified impact of the Pleistocene sea level and salinity fluctuations on the phylogeographic structure of *Dikerogammarus villosus* in the Black Sea native region and presence of two differentiated source populations, i.e. the Danube and Dnieper deltas. These sources are associated with two independent invasion routes (Western and Eastern) in continental Europe for which no loss of diversity is observed. We can expect further spread of the killer shrimp in continental Europe, even in smaller tributaries. MacNeil & Platvoet [95] pointed out that solid objects, like concrete fish passages, could be used by *D. villosus* as mainstay in smaller tributaries. This may pose a threat to native gammarids occupying such refuges [109].

The UK population has probably originated in the Western Route and despite very recent colonization, no drastic loss of diversity was observed. This recent overseas conquest provides rather non-optimistic message, accounting that the UK authorities implemented preventive biosecurity protocols and risk assessments. The Great Lakes of North America are likely to be the next step, since other Ponto-Caspian invertebrates already managed to reach them [110, 111].

Finally, the Dniester native area is characterized by high msat allelic diversity (including private alleles). Thus, even if at the moment it is not a source population for the colonization of Europe, it may act as a donor for other source areas if the anthropogenic transport increases, enhancing the local genetic diversity.

## Supporting Information

S1 Table GeneBank accession numbers of COI and 16S haplotypes. (DOCX)
