# Supplementary material for: Out of the Black Sea: Phylogeography of the Invasive Killer Shrimp Dikerogammarus villosus across Europe
Source: PLoS One. 2015 Feb 18;10(2):e0118121. doi: 10.1371/journal.pone.0118121 (PMC4333216; doi:10.1371/journal.pone.0118121)
Supplement: S1 Table — (DOCX) [file pone.0118121.s001.docx]

| Individuals | 16S | COI | concatenated 16S and COI |
| --- | --- | --- | --- |
| 1-N/1 | hap 1 KM208876 | hap 1 KM208862 | hap 1 |
| 1-N/10 | hap 1 KM208876 | hap 1 KM208862 | hap 1 |
| 1-N/11 | hap 1 KM208876 | hap 1 KM208862 | hap 1 |
| 1-N/12 | hap 1 KM208876 | hap 1 KM208862 | hap 2 |
| 1-N/2 | hap 1 KM208876 | hap 2 KM208863 | hap 2 |
| 1-N/3 | hap 1 KM208876 | hap 2 KM208863 | hap 2 |
| 1-N/30 | hap 1 KM208876 | hap 3 KM208864 | hap 3 |
| 1-N/31 | hap 1 KM208876 | hap 1 KM208862 | hap 1 |
| 1-N/32 | hap 1 KM208876 | hap 2 KM208863 | hap 2 |
| 1-N/33 | hap 1 KM208876 | hap 4 KM208865 | hap 4 |
| 1-N/34 | hap 1 KM208876 | hap 2 KM208863 | hap 2 |
| 1-N/35 | hap 1 KM208876 | hap 1 KM208862 | hap 1 |
| 1-N/36 | hap 1 KM208876 | hap 1 KM208862 | hap 1 |
| 1-N/37 | hap 1 KM208876 | hap 2 KM208863 | hap 2 |
| 1-N/38 | hap 1 KM208876 | hap 1 KM208862 | hap 1 |
| 1-N/39 | hap 1 KM208876 | hap 2 KM208863 | hap 2 |
| 1-N/4 | hap 1 KM208876 | hap 2 KM208863 | hap 2 |
| 1-N/40 | hap 1 KM208876 | hap 2 KM208863 | hap 2 |
| 1-N/41 | hap 1 KM208876 | hap 2 KM208863 | hap 2 |
| 1-N/5 | hap 1 KM208876 | hap 2 KM208863 | hap 2 |
| 1-N/6 | hap 1 KM208876 | hap 1 KM208862 | hap 1 |
| 1-N/7 | hap 1 KM208876 | hap 2 KM208863 | hap 2 |
| 1-N/8 | hap 1 KM208876 | hap 2 KM208863 | hap 2 |
| 1-N/9 | hap 1 KM208876 | hap 2 KM208863 | hap 2 |
| 2-N/1 | hap 1 KM208876 | hap 5 KM208866 | hap 5 |
| 2-N/10 | hap 1 KM208876 | hap 5 KM208866 | hap 5 |
| 2-N/11 | hap 1 KM208876 | hap 5 KM208866 | hap 5 |
| 2-N/12 | hap 1 KM208876 | hap 6 KM208867 | hap 6 |
| 2-N/2 | hap 1 KM208876 | hap 7 KM208868 | hap 7 |
| 2-N/3 | hap 1 KM208876 | hap 5 KM208866 | hap 5 |
| 2-N/30 | hap 1 KM208876 | hap 8 KM208869 | hap 8 |
| 2-N/31 | hap 1 KM208876 | hap 5 KM208866 | hap 5 |
| 2-N/32 | hap 1 KM208876 | hap 5 KM208866 | hap 5 |
| 2-N/33 | hap 1 KM208876 | hap 5 KM208866 | hap 5 |
| 2-N/34 | hap 1 KM208876 | hap 5 KM208866 | hap 5 |
| 2-N/35 | hap 1 KM208876 | hap 5 KM208866 | hap 5 |
| 2-N/36 | hap 1 KM208876 | hap 5 KM208866 | hap 5 |
| 2-N/37 | hap 1 KM208876 | hap 9 KM208870 | hap 9 |
| 2-N/38 | hap 1 KM208876 | hap 5 KM208866 | hap 5 |
| 2-N/39 | hap 1 KM208876 | hap 10 KM208871 | hap 10 |
| 2-N/4 | hap 1 KM208876 | hap 10 KM208871 | hap 10 |
| 2-N/41 | hap 1 KM208876 | hap 5 KM208866 | hap 5 |
| 2-N/5 | hap 1 KM208876 | hap 5 KM208866 | hap 5 |
| 2-N/6 | hap 2 KM208877 | hap 5 KM208866 | hap 11 |
| 2-N/7 | hap 1 KM208876 | hap 5 KM208866 | hap 5 |
| 2-N/8 | hap 3 KM208878 | hap 5 KM208866 | hap 12 |
| 2-N/9 | hap 1 KM208876 | hap 5 KM208866 | hap 5 |
| 3-N/1 | hap 1 KM208876 | hap 1 KM208862 | hap 1 |
| 3-N/10 | hap 1 KM208876 | hap 1 KM208862 | hap 1 |
| 3-N/11 | hap 1 KM208876 | hap 1 KM208862 | hap 1 |
| 3-N/12 | hap 1 KM208876 | hap 1 KM208862 | hap 1 |
| 3-N/2 | hap 1 KM208876 | hap 1 KM208862 | hap 1 |
| 3-N/3 | hap 1 KM208876 | hap 1 KM208862 | hap 1 |
| 3-N/30 | hap 1 KM208876 | hap 1 KM208862 | hap 1 |
| 3-N/4 | hap 1 KM208876 | hap 11 KM208872 | hap 13 |
| 3-N/5 | hap 1 KM208876 | hap 1 KM208862 | hap 1 |
| 3-N/6 | hap 1 KM208876 | hap 1 KM208862 | hap 1 |
| 3-N/7 | hap 1 KM208876 | hap 1 KM208862 | hap 1 |
| 3-N/8 | hap 1 KM208876 | hap 1 KM208862 | hap 1 |
| 3-N/9 | hap 1 KM208876 | hap 1 KM208862 | hap 1 |
| 3-N/13 | hap 1 KM208876 | hap 1 KM208862 | hap 1 |
| 3-N/14 | hap 1 KM208876 | hap 1 KM208862 | hap 1 |
| 3-N/15 | hap 1 KM208876 | hap 1 KM208862 | hap 1 |
| 3-N/16 | hap 1 KM208876 | hap 1 KM208862 | hap 1 |
| 3-N/17 | hap 1 KM208876 | hap 11 KM208872 | hap 13 |
| 4-N/1 | hap 1 KM208876 | hap 11 KM208872 | hap 13 |
| 4-N/10 | hap 1 KM208876 | hap 1 KM208862 | hap 1 |
| 4-N/11 | hap 1 KM208876 | hap 11 KM208872 | hap 13 |
| 4-N/12 | hap 1 KM208876 | hap 1 KM208862 | hap 1 |
| 4-N/2 | hap 1 KM208876 | hap 11 KM208872 | hap 13 |
| 4-N/3 | hap 1 KM208876 | hap 1 KM208862 | hap 1 |
| 4-N/30 | hap 1 KM208876 | hap 1 KM208862 | hap 1 |
| 4-N/4 | hap 1 KM208876 | hap 1 KM208862 | hap 1 |
| 4-N/5 | hap 1 KM208876 | hap 11 KM208872 | hap 13 |
| 4-N/6 | hap 1 KM208876 | hap 11 KM208872 | hap 13 |
| 4-N/7 | hap 1 KM208876 | hap 1 KM208862 | hap 1 |
| 4-N/8 | hap 1 KM208876 | hap 11 KM208872 | hap 13 |
| 4-N/9 | hap 1 KM208876 | hap 11 KM208872 | hap 13 |
| 5-N/1 | hap 1 KM208876 | hap 1 KM208862 | hap 1 |
| 5-N/10 | hap 1 KM208876 | hap 1 KM208862 | hap 1 |
| 5-N/11 | hap 1 KM208876 | hap 1 KM208862 | hap 1 |
| 5-N/13 | hap 1 KM208876 | hap 1 KM208862 | hap 1 |
| 5-N/14 | hap 1 KM208876 | hap 1 KM208862 | hap 1 |
| 5-N/16 | hap 1 KM208876 | hap 1 KM208862 | hap 1 |
| 5-N/17 | hap 1 KM208876 | hap 1 KM208862 | hap 1 |
| 5-N/18 | hap 1 KM208876 | hap 1 KM208862 | hap 1 |
| 5-N/19 | hap 1 KM208876 | hap 1 KM208862 | hap 1 |
| 5-N/21 | hap 1 KM208876 | hap 1 KM208862 | hap 1 |
| 5-N/4 | hap 1 KM208876 | hap 1 KM208862 | hap 1 |
| 6-N/1 | hap 1 KM208876 | hap 1 KM208862 | hap 1 |
| 6-N/10 | hap 1 KM208876 | hap 1 KM208862 | hap 1 |
| 6-N/12 | hap 1 KM208876 | hap 1 KM208862 | hap 1 |
| 6-N/13 | hap 1 KM208876 | hap 1 KM208862 | hap 1 |
| 6-N/2 | hap 1 KM208876 | hap 1 KM208862 | hap 1 |
| 6-N/3 | hap 1 KM208876 | hap 1 KM208862 | hap 1 |
| 6-N/4 | hap 1 KM208876 | hap 1 KM208862 | hap 1 |
| 6-N/5 | hap 1 KM208876 | hap 1 KM208862 | hap 1 |
| 6-N/6 | hap 1 KM208876 | hap 1 KM208862 | hap 1 |
| 6-N/7 | hap 1 KM208876 | hap 1 KM208862 | hap 1 |
| 6-N/8 | hap 1 KM208876 | hap 1 KM208862 | hap 1 |
| 6-N/9 | hap 1 KM208876 | hap 1 KM208862 | hap 1 |
| 7-N/1 | hap 1 KM208876 | hap 1 KM208862 | hap 1 |
| 7-N/10 | hap 1 KM208876 | hap 12 KM208873 | hap 14 |
| 7-N/11 | hap 1 KM208876 | hap 1 KM208862 | hap 1 |
| 7-N/13 | hap 1 KM208876 | hap 2 KM208863 | hap 2 |
| 7-N/14 | hap 1 KM208876 | hap 1 KM208862 | hap 1 |
| 7-N/16 | hap 1 KM208876 | hap 1 KM208862 | hap 1 |
| 7-N/3 | hap 1 KM208876 | hap 1 KM208862 | hap 1 |
| 7-N/4 | hap 1 KM208876 | hap 1 KM208862 | hap 1 |
| 7-N/5 | hap 1 KM208876 | hap 1 KM208862 | hap 1 |
| 7-N/6 | hap 1 KM208876 | hap 1 KM208862 | hap 1 |
| 7-N/8 | hap 1 KM208876 | hap 12 KM208873 | hap 14 |
| 7-N/9 | hap 1 KM208876 | hap 12 KM208873 | hap 14 |
| 8-N/12 | hap 1 KM208876 | hap 1 KM208862 | hap 1 |
| 8-N/50 | hap 1 KM208876 | hap 1 KM208862 | hap 1 |
| 8-N/52 | hap 4 KM208879 | hap 13 KM208874 | hap 15 |
| 8-N/53 | hap 3 KM208878 | hap 1 KM208862 | hap 16 |
| 8-N/54 | hap 1 KM208876 | hap 1 KM208862 | hap 1 |
| 8-N/55 | hap 1 KM208876 | hap 1 KM208862 | hap 1 |
| 8-N/56 | hap 1 KM208876 | hap 1 KM208862 | hap 1 |
| 8-N/57 | hap 1 KM208876 | hap 1 KM208862 | hap 1 |
| 8-N/65 | hap 1 KM208876 | hap 1 KM208862 | hap 1 |
| 8-N/84 | hap 1 KM208876 | hap 1 KM208862 | hap 1 |
| 9-N/10 | hap 1 KM208876 | hap 1 KM208862 | hap 1 |
| 9-N/13 | hap 1 KM208876 | hap 14 KM208875 | hap 17 |
| 9-N/17 | hap 1 KM208876 | hap 1 KM208862 | hap 1 |
| 9-N/19 | hap 1 KM208876 | hap 1 KM208862 | hap 1 |
| 9-N/27 | hap 1 KM208876 | hap 1 KM208862 | hap 1 |
| 9-N/28 | hap 1 KM208876 | hap 1 KM208862 | hap 1 |
| 9-N/32 | hap 1 KM208876 | hap 1 KM208862 | hap 1 |
| 9-N/35 | hap 1 KM208876 | hap 1 KM208862 | hap 1 |
| 9-N/5 | hap 1 KM208876 | hap 1 KM208862 | hap 1 |
| 9-N/7 | hap 1 KM208876 | hap 1 KM208862 | hap 1 |
| 10-ER/17 | hap 1 KM208876 | hap 1 KM208862 | hap 1 |
| 10-ER/23 | hap 1 KM208876 | hap 1 KM208862 | hap 1 |
| 10-ER/25 | hap 1 KM208876 | hap 1 KM208862 | hap 1 |
| 10-ER/32 | hap 1 KM208876 | hap 1 KM208862 | hap 1 |
| 10-ER/37 | hap 1 KM208876 | hap 11 KM208872 | hap 13 |
| 10-ER/38 | hap 1 KM208876 | hap 11 KM208872 | hap 13 |
| 10-ER/40 | hap 1 KM208876 | hap 11 KM208872 | hap 13 |
| 10-ER/53 | hap 1 KM208876 | hap 1 KM208862 | hap 1 |
| 10-ER/54 | hap 1 KM208876 | hap 1 KM208862 | hap 1 |
| 10-ER/57 | hap 1 KM208876 | hap 11 KM208872 | hap 13 |
| 10-ER/59 | hap 1 KM208876 | hap 11 KM208872 | hap 13 |
| 10-ER/66 | hap 1 KM208876 | hap 1 KM208862 | hap 1 |
| 10-ER/78 | hap 1 KM208876 | hap 1 KM208862 | hap 1 |
| 11-ER/10 | hap 1 KM208876 | hap 11 KM208872 | hap 13 |
| 11-ER/13 | hap 1 KM208876 | hap 1 KM208862 | hap 1 |
| 11-ER/28 | hap 1 KM208876 | hap 1 KM208862 | hap 1 |
| 11-ER/32 | hap 1 KM208876 | hap 11 KM208872 | hap 13 |
| 11-ER/4 | hap 1 KM208876 | hap 1 KM208862 | hap 1 |
| 12-WR/10 | hap 1 KM208876 | hap 1 KM208862 | hap 1 |
| 12-WR/16 | hap 1 KM208876 | hap 1 KM208862 | hap 1 |
| 12-WR/17 | hap 3 KM208878 | hap 1 KM208862 | hap 16 |
| 12-WR/20 | hap 1 KM208876 | hap 1 KM208862 | hap 1 |
| 12-WR/21 | hap 1 KM208876 | hap 2 KM208863 | hap 2 |
| 12-WR/25 | hap 1 KM208876 | hap 1 KM208862 | hap 1 |
| 12-WR/27 | hap 1 KM208876 | hap 1 KM208862 | hap 1 |
| 12-WR/28 | hap 1 KM208876 | hap 1 KM208862 | hap 1 |
| 12-WR/3 | hap 1 KM208876 | hap 1 KM208862 | hap 1 |
| 12-WR/4 | hap 1 KM208876 | hap 1 KM208862 | hap 1 |
| 12-WR/5 | hap 1 KM208876 | hap 1 KM208862 | hap 1 |
| 12-WR/9 | hap 1 KM208876 | hap 1 KM208862 | hap 1 |
| 13-WR/10 | hap 1 KM208876 | hap 1 KM208862 | hap 1 |
| 13-WR/36 | hap 1 KM208876 | hap 1 KM208862 | hap 1 |
| 13-WR/37 | hap 1 KM208876 | hap 1 KM208862 | hap 1 |
| 13-WR/4 | hap 1 KM208876 | hap 1 KM208862 | hap 1 |
| 13-WR/42 | hap 1 KM208876 | hap 1 KM208862 | hap 1 |
| 13-WR/48 | hap 1 KM208876 | hap 1 KM208862 | hap 1 |
| 14-WR/1 | hap 1 KM208876 | hap 1 KM208862 | hap 1 |
| 14-WR/10 | hap 1 KM208876 | hap 1 KM208862 | hap 1 |
| 14-WR/11 | hap 1 KM208876 | hap 1 KM208862 | hap 1 |
| 14-WR/12 | hap 1 KM208876 | hap 14 KM208875 | hap 17 |
| 14-WR/13 | hap 1 KM208876 | hap 14 KM208875 | hap 17 |
| 14-WR/3 | hap 1 KM208876 | hap 1 KM208862 | hap 1 |
| 14-WR/4 | hap 1 KM208876 | hap 1 KM208862 | hap 1 |
| 14-WR/5 | hap 1 KM208876 | hap 1 KM208862 | hap 1 |
| 14-WR/6 | hap 1 KM208876 | hap 14 KM208875 | hap 17 |
| 14-WR/7 | hap 1 KM208876 | hap 1 KM208862 | hap 1 |
| 14-WR/8 | hap 1 KM208876 | hap 1 KM208862 | hap 1 |
| 14-WR/9 | hap 1 KM208876 | hap 14 KM208875 | hap 17 |
| 15-WR/10 | hap 1 KM208876 | hap 1 KM208862 | hap 1 |
| 15-WR/11 | hap 1 KM208876 | hap 1 KM208862 | hap 1 |
| 15-WR/12 | hap 1 KM208876 | hap 1 KM208862 | hap 1 |
| 15-WR/2 | hap 3 KM208878 | hap 1 KM208862 | hap 16 |
| 15-WR/3 | hap 4 KM208879 | hap 13 KM208874 | hap 15 |
| 15-WR/4 | hap 1 KM208876 | hap 1 KM208862 | hap 1 |
| 15-WR/5 | hap 1 KM208876 | hap 1 KM208862 | hap 1 |
| 15-WR/6 | hap 1 KM208876 | hap 1 KM208862 | hap 1 |
| 15-WR/7 | hap 1 KM208876 | hap 1 KM208862 | hap 1 |
| 15-WR/8 | hap 1 KM208876 | hap 1 KM208862 | hap 1 |
| 15-WR/9 | hap 4 KM208879 | hap 13 KM208874 | hap 15 |
| 16-WR/15 | hap 1 KM208876 | hap 1 KM208862 | hap 1 |
| 16-WR/17 | hap 1 KM208876 | hap 1 KM208862 | hap 1 |
| 16-WR/2 | hap 1 KM208876 | hap 1 KM208862 | hap 1 |
| 16-WR/22 | hap 1 KM208876 | hap 1 KM208862 | hap 1 |
| 16-WR/25 | hap 1 KM208876 | hap 1 KM208862 | hap 1 |
| 16-WR/28 | hap 1 KM208876 | hap 1 KM208862 | hap 1 |
| 16-WR/35 | hap 1 KM208876 | hap 1 KM208862 | hap 1 |
| 16-WR/4 | hap 1 KM208876 | hap 1 KM208862 | hap 1 |
| 16-WR/6 | hap 1 KM208876 | hap 1 KM208862 | hap 1 |
| 16-WR/7 | hap 1 KM208876 | hap 1 KM208862 | hap 1 |
| 17-WR/1 | hap 1 KM208876 | hap 1 KM208862 | hap 1 |
| 17-WR/20 | hap 1 KM208876 | hap 1 KM208862 | hap 1 |
| 17-WR/29 | hap 1 KM208876 | hap 1 KM208862 | hap 1 |
| 17-WR/39 | hap 1 KM208876 | hap 1 KM208862 | hap 1 |
| 17-WR/43 | hap 1 KM208876 | hap 1 KM208862 | hap 1 |
| 17-WR/9 | hap 1 KM208876 | hap 1 KM208862 | hap 1 |
| 18-WR/2 | hap 1 KM208876 | hap 1 KM208862 | hap 1 |
| 18-WR/23 | hap 1 KM208876 | hap 1 KM208862 | hap 1 |
| 18-WR/26 | hap 1 KM208876 | hap 1 KM208862 | hap 1 |
| 18-WR/31 | hap 1 KM208876 | hap 1 KM208862 | hap 1 |
| 18-WR/37 | hap 1 KM208876 | hap 1 KM208862 | hap 1 |
| 18-WR/4 | hap 1 KM208876 | hap 1 KM208862 | hap 1 |
| 18-WR/43 | hap 4 KM208879 | hap 13 KM208874 | hap 15 |
| 19-WR/15 | hap 1 KM208876 | hap 1 KM208862 | hap 1 |
| 19-WR/16 | hap 1 KM208876 | hap 1 KM208862 | hap 1 |
| 19-WR/17 | hap 1 KM208876 | hap 1 KM208862 | hap 1 |
| 19-WR/20 | hap 1 KM208876 | hap 1 KM208862 | hap 1 |
| 19-WR/21 | hap 1 KM208876 | hap 1 KM208862 | hap 1 |
| 19-WR/22 | hap 1 KM208876 | hap 1 KM208862 | hap 1 |
| 20-WR/18 | hap 1 KM208876 | hap 1 KM208862 | hap 1 |
| 20-WR/2 | hap 1 KM208876 | hap 1 KM208862 | hap 1 |
| 20-WR/5 | hap 1 KM208876 | hap 1 KM208862 | hap 1 |
| 20-WR/6 | hap 1 KM208876 | hap 1 KM208862 | hap 1 |
| 20-WR/7 | hap 1 KM208876 | hap 1 KM208862 | hap 1 |
| 20-WR/9 | hap 1 KM208876 | hap 1 KM208862 | hap 1 |
| 21-WR/12 | hap 1 KM208876 | hap 1 KM208862 | hap 1 |
| 21-WR/21 | hap 1 KM208876 | hap 1 KM208862 | hap 1 |
| 21-WR/25 | hap 1 KM208876 | hap 2 KM208863 | hap 2 |
| 21-WR/26 | hap 1 KM208876 | hap 1 KM208862 | hap 1 |
| 21-WR/4 | hap 1 KM208876 | hap 1 KM208862 | hap 1 |
| 21-WR/5 | hap 1 KM208876 | hap 1 KM208862 | hap 1 |
| 22-WR/11 | hap 1 KM208876 | hap 2 KM208863 | hap 2 |
| 22-WR/13 | hap 1 KM208876 | hap 14 KM208875 | hap 17 |
| 22-WR/14 | hap 1 KM208876 | hap 1 KM208862 | hap 1 |
| 22-WR/2 | hap 1 KM208876 | hap 14 KM208875 | hap 17 |
| 22-WR/4 | hap 1 KM208876 | hap 1 KM208862 | hap 1 |
| 22-WR/5 | hap 1 KM208876 | hap 1 KM208862 | hap 1 |
| 23-WR/1 | hap 1 KM208876 | hap 1 KM208862 | hap 1 |
| 23-WR/10 | hap 1 KM208876 | hap 1 KM208862 | hap 1 |
| 23-WR/11 | hap 1 KM208876 | hap 1 KM208862 | hap 1 |
| 23-WR/12 | hap 1 KM208876 | hap 1 KM208862 | hap 1 |
| 23-WR/13 | hap 1 KM208876 | hap 1 KM208862 | hap 1 |
| 23-WR/3 | hap 1 KM208876 | hap 1 KM208862 | hap 1 |
| 23-WR/4 | hap 1 KM208876 | hap 1 KM208862 | hap 1 |
| 23-WR/5 | hap 1 KM208876 | hap 1 KM208862 | hap 1 |
| 23-WR/6 | hap 4 KM208879 | hap 13 KM208874 | hap 15 |
| 23-WR/7 | hap 1 KM208876 | hap 1 KM208862 | hap 1 |
| 23-WR/8 | hap 1 KM208876 | hap 1 KM208862 | hap 1 |
| 23-WR/9 | hap 1 KM208876 | hap 1 KM208862 | hap 1 |
| 24-WR/17 | hap 1 KM208876 | hap 1 KM208862 | hap 1 |
| 24-WR/31 | hap 1 KM208876 | hap 1 KM208862 | hap 1 |
| 24-WR/37 | hap 1 KM208876 | hap 12 KM208873 | hap 14 |
| 24-WR/38 | hap 1 KM208876 | hap 1 KM208862 | hap 1 |
| 24-WR/42 | hap 1 KM208876 | hap 1 KM208862 | hap 1 |
| 24-WR/43 | hap 1 KM208876 | hap 1 KM208862 | hap 1 |
| 25-WR/11 | hap 1 KM208876 | hap 1 KM208862 | hap 1 |
| 25-WR/15 | hap 1 KM208876 | hap 1 KM208862 | hap 1 |
| 25-WR/19 | hap 1 KM208876 | hap 1 KM208862 | hap 1 |
| 25-WR/3 | hap 1 KM208876 | hap 1 KM208862 | hap 1 |
| 25-WR/4 | hap 1 KM208876 | hap 1 KM208862 | hap 1 |
| 26-WR/1 | hap 1 KM208876 | hap 1 KM208862 | hap 1 |
| 26-WR/14 | hap 1 KM208876 | hap 1 KM208862 | hap 1 |
| 26-WR/15 | hap 1 KM208876 | hap 1 KM208862 | hap 1 |
| 26-WR/17 | hap 1 KM208876 | hap 1 KM208862 | hap 1 |
| 26-WR/5 | hap 1 KM208876 | hap 1 KM208862 | hap 1 |
| 26-WR/9 | hap 1 KM208876 | hap 1 KM208862 | hap 1 |
| 27-WR/32 | hap 1 KM208876 | hap 1 KM208862 | hap 1 |
| 27-WR/35 | hap 1 KM208876 | hap 1 KM208862 | hap 1 |
| 27-WR/38 | hap 1 KM208876 | hap 1 KM208862 | hap 1 |
| 27-WR/39 | hap 1 KM208876 | hap 1 KM208862 | hap 1 |
| 27-WR/4 | hap 1 KM208876 | hap 1 KM208862 | hap 1 |
| 27-WR/42 | hap 3 KM208878 | hap 1 KM208862 | hap 16 |
| 28-WR/10 | hap 1 KM208876 | hap 2 KM208863 | hap 2 |
| 28-WR/11 | hap 1 KM208876 | hap 2 KM208863 | hap 2 |
| 28-WR/12 | hap 1 KM208876 | hap 2 KM208863 | hap 2 |
| 28-WR/13 | hap 1 KM208876 | hap 13 KM208874 | hap 18 |
| 28-WR/14 | hap 1 KM208876 | hap 2 KM208863 | hap 2 |
| 28-WR/19 | hap 1 KM208876 | hap 2 KM208863 | hap 2 |
| 28-WR/2 | hap 1 KM208876 | hap 1 KM208862 | hap 1 |
| 28-WR/3 | hap 1 KM208876 | hap 1 KM208862 | hap 1 |
| 28-WR/6 | hap 1 KM208876 | hap 2 KM208863 | hap 2 |
| 28-WR/7 | hap 1 KM208876 | hap 1 KM208862 | hap 1 |
| 28-WR/8 | hap 1 KM208876 | hap 1 KM208862 | hap 1 |
| 28-WR/9 | hap 1 KM208876 | hap 1 KM208862 | hap 1 |
| 29-WR/1 | hap 1 KM208876 | hap 2 KM208863 | hap 2 |
| 29-WR/10 | hap 1 KM208876 | hap 1 KM208862 | hap 1 |
| 29-WR/12 | hap 1 KM208876 | hap 2 KM208863 | hap 2 |
| 29-WR/14 | hap 1 KM208876 | hap 2 KM208863 | hap 2 |
| 29-WR/16 | hap 1 KM208876 | hap 1 KM208862 | hap 1 |
| 29-WR/17 | hap 1 KM208876 | hap 1 KM208862 | hap 1 |
| 29-WR/19 | hap 1 KM208876 | hap 1 KM208862 | hap 1 |
| 29-WR/20 | hap 1 KM208876 | hap 1 KM208862 | hap 1 |
| 29-WR/3 | hap 1 KM208876 | hap 1 KM208862 | hap 1 |
| 29-WR/5 | hap 1 KM208876 | hap 1 KM208862 | hap 1 |
| 29-WR/7 | hap 1 KM208876 | hap 1 KM208862 | hap 1 |
| 29-WR/9 | hap 1 KM208876 | hap 2 KM208863 | hap 2 |
| 30-WR/1 | hap 1 KM208876 | hap 1 KM208862 | hap 1 |
| 30-WR/10 | hap 1 KM208876 | hap 1 KM208862 | hap 1 |
| 30-WR/11 | hap 1 KM208876 | hap 1 KM208862 | hap 1 |
| 30-WR/12 | hap 1 KM208876 | hap 1 KM208862 | hap 1 |
| 30-WR/2 | hap 1 KM208876 | hap 1 KM208862 | hap 1 |
| 30-WR/3 | hap 1 KM208876 | hap 1 KM208862 | hap 1 |
| 30-WR/4 | hap 1 KM208876 | hap 1 KM208862 | hap 1 |
| 30-WR/5 | hap 4 KM208879 | hap 13 KM208874 | hap 15 |
| 30-WR/6 | hap 1 KM208876 | hap 1 KM208862 | hap 1 |
| 30-WR/7 | hap 1 KM208876 | hap 1 KM208862 | hap 1 |
| 30-WR/8 | hap 1 KM208876 | hap 1 KM208862 | hap 1 |
| 30-WR/9 | hap 1 KM208876 | hap 1 KM208862 | hap 1 |
| 31-WR/13 | hap 1 KM208876 | hap 1 KM208862 | hap 1 |
| 31-WR/14 | hap 1 KM208876 | hap 1 KM208862 | hap 1 |
| 31-WR/15 | hap 1 KM208876 | hap 1 KM208862 | hap 1 |
| 31-WR/16 | hap 1 KM208876 | hap 14 KM208875 | hap 17 |
| 31-WR/17 | hap 1 KM208876 | hap 1 KM208862 | hap 1 |
| 31-WR/19 | hap 1 KM208876 | hap 1 KM208862 | hap 1 |
| 31-WR/20 | hap 1 KM208876 | hap 1 KM208862 | hap 1 |
| 31-WR/21 | hap 1 KM208876 | hap 1 KM208862 | hap 1 |
| 31-WR/22 | hap 1 KM208876 | hap 1 KM208862 | hap 1 |
| 31-WR/24 | hap 1 KM208876 | hap 1 KM208862 | hap 1 |
| 31-WR/9 | hap 1 KM208876 | hap 1 KM208862 | hap 1 |
| 32A-UK/10 | hap 1 KM208876 | hap 1 KM208862 | hap 1 |
| 32A-UK/11 | hap 1 KM208876 | hap 1 KM208862 | hap 1 |
| 32A-UK/14 | hap 1 KM208876 | hap 1 KM208862 | hap 1 |
| 32A-UK/15 | hap 1 KM208876 | hap 1 KM208862 | hap 1 |
| 32A-UK/17 | hap 1 KM208876 | hap 1 KM208862 | hap 1 |
| 32A-UK/18 | hap 1 KM208876 | hap 1 KM208862 | hap 1 |
| 32A-UK/2 | hap 1 KM208876 | hap 1 KM208862 | hap 1 |
| 32A-UK/20 | hap 1 KM208876 | hap 1 KM208862 | hap 1 |
| 32A-UK/3 | hap 1 KM208876 | hap 1 KM208862 | hap 1 |
| 32A-UK/5 | hap 1 KM208876 | hap 1 KM208862 | hap 1 |
| 32A-UK/7 | hap 1 KM208876 | hap 1 KM208862 | hap 1 |
| 32A-UK/9 | hap 1 KM208876 | hap 1 KM208862 | hap 1 |
| 32B-UK/10 | hap 1 KM208876 | hap 1 KM208862 | hap 1 |
| 32B-UK/12 | hap 1 KM208876 | hap 1 KM208862 | hap 1 |
| 32B-UK/14 | hap 1 KM208876 | hap 1 KM208862 | hap 1 |
| 32B-UK/4 | hap 1 KM208876 | hap 1 KM208862 | hap 1 |
| 32B-UK/5 | hap 1 KM208876 | hap 1 KM208862 | hap 1 |
| 32B-UK/6 | hap 1 KM208876 | hap 1 KM208862 | hap 1 |
| 32B-UK/7 | hap 1 KM208876 | hap 1 KM208862 | hap 1 |
| 32B-UK/8 | hap 1 KM208876 | hap 1 KM208862 | hap 1 |
| 32B-UK/9 | hap 1 KM208876 | hap 1 KM208862 | hap 1 |
| 33-UK/1 | hap 1 KM208876 | hap 1 KM208862 | hap 1 |
| 33-UK/2 | hap 1 KM208876 | hap 1 KM208862 | hap 1 |
| 33-UK/3 | hap 1 KM208876 | hap 1 KM208862 | hap 1 |
| 33-UK/4 | hap 1 KM208876 | hap 1 KM208862 | hap 1 |
| 33-UK/5 | hap 1 KM208876 | hap 1 KM208862 | hap 1 |
| 33-UK/6 | hap 1 KM208876 | hap 1 KM208862 | hap 1 |
| 33-UK/7 | hap 1 KM208876 | hap 1 KM208862 | hap 1 |
| 33-UK/8 | hap 1 KM208876 | hap 1 KM208862 | hap 1 |
